# Why is it so hard to reduce harm from medicines?

**DOI:** 10.1016/j.fhj.2024.100205

**Published:** 2024-12-12

**Authors:** Andrew Rochford

**Affiliations:** Consultant Gastroenterologist, Royal Free London NHS Foundation Trust, Improvement Clinical Director, Royal College of Physicians, Chair, Royal Colleges Medicines Safety Joint Working Group, Care Quality Improvement Directorate, 11 St Andrews Place, Regent's Park, London, NW1 4LE, UK

**Keywords:** Medication safety, Patient safety, Medication errors

## Abstract

Pharmacotherapy is the most common therapeutic intervention in healthcare, but more than 200 million medication errors occur every year in England alone. This may in part reflect greater awareness and better reporting; however, the incidence of patient harm from medication has remained broadly unchanged for decades, despite concerted national campaigns and global safety initiatives. Rapid technological and therapeutic advances together with the complexity of modern healthcare make reducing harm from medicines more challenging than ever. This opinion piece will provide a perspective on some of those challenges, as well as highlighting areas of best practice and emerging work. While system and process improvements are required, individual clinicians need to remain vigilant and reflect on medications at each patient interaction.

## Introduction

Pharmacotherapy is the most common therapeutic intervention in healthcare. The World Health Organization (WHO) recognises that ‘medicines do sometimes cause serious harm if taken incorrectly, monitored insufficiently or as the result of an error, accident or communication problem’. Experience from other high-risk industries demonstrates that humans rarely make mistakes through neglect, but instead because the systems, processes and procedures that they work with are often flawed or dysfunctional. This inevitably gives rise to errors, and medication harm is no exception to this rule. All medication errors (MEs) are potentially avoidable.^1^

Healthcare is recognised as a complex adaptive system and the challenges that this poses for medicines safety have been well studied. >200 million MEs occur every year in England, which makes me reflect on Sir Cyril Chantler’s statement that ‘medicine used to be simple, ineffective, and relatively safe. It is now complex, effective, and potentially dangerous’.

The Royal Colleges Medicines Safety Joint Working Group (MSJWG) was established in response to the WHO Global Patient Safety Challenge: Medication Without Harm and brings together key UK healthcare organisations and experts for whom the safe use of medicines is a central part of professional practice. As chair of the MSJWG, I have the privilege of seeing the breadth of work that is happening to improve medicines safety in the NHS and beyond; however, as a practising clinician I worry about patient safety and MEs on a daily basis, and wonder why this feels so difficult.

There have undoubtedly been positive changes both in terms of systems, processes and people, but these seem to have been eclipsed by the increasing challenges that are faced. The MSJWG brings together professionals working to address those challenges.

## Healthcare provision

**Fig. 1:**
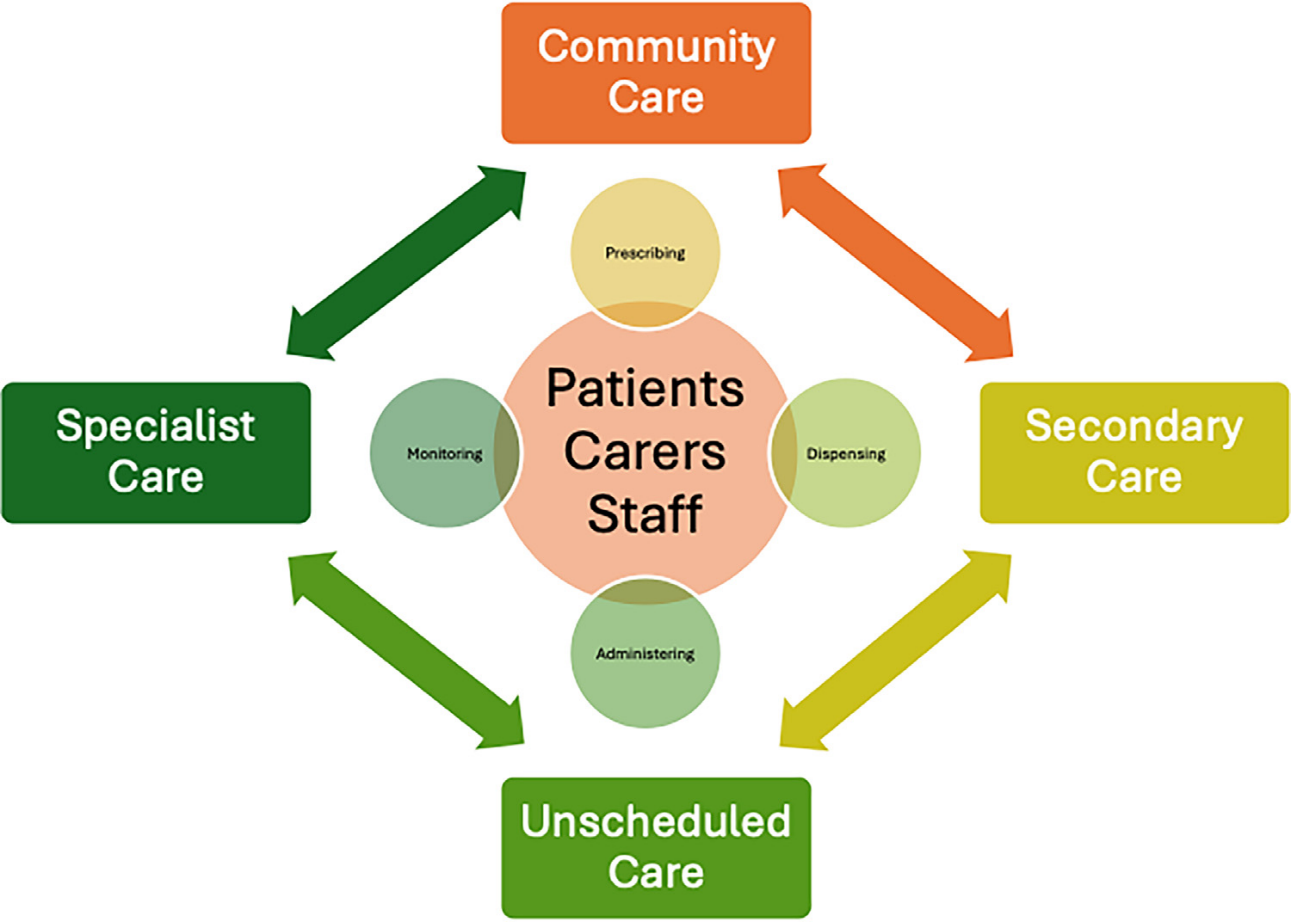
Medication safety and transitions of care. Adapted from Donaldson L, et al.^2^

The landscape for healthcare provision has never been so diverse or confusing for both patients and professionals (see Fig. 1). In addition to this there is also private care provision, including the widespread availability of medications online. Each healthcare interaction can introduce MEs and this has been well studied within medication administration and monitoring processes, as well as in the transition between locations of care.

In 2005, a quarter of hospital prescribing errors were attributed to incomplete medication histories obtained at the time of admission^3^ which led to the introduction of ‘medication reconciliation’. Despite this, a national multi-site audit in 2016 highlighted that three in ten patients had unintentional omissions of preadmission medication.^4^ MEs can be perpetuated within hospital as well as at discharge, ranging from a simple lack of documentation of medication changes to omission of chronic disease medication and/or continuation of inappropriate acute medication. MEs are particularly challenging when transitioning patients from intensive care units to wards, with MEs reported to occur in between 46% and 74% of patients.^5^

## New medicines, polypharmacy and multimorbidity

When I began specialist training 20 years ago, we started to use a ‘new’ anti-tumour necrosis factor monoclonal antibody called adalimumab. Patients attended clinic fortnightly for the subcutaneous injection; the medication is now delivered directly to patients at home, where patients are taught to self-administer. There are currently eight ‘biosimilars’ of adalimumab available in Europe and at least 21 novel drug therapies in the development pipeline for the treatment of inflammatory bowel disease (IBD). Staying up to date as a specialist is extremely challenging, even more so for a generalist.

An ageing population and the rising prevalence of multimorbidity has led to increasing polypharmacy. It is probably best considered as appropriate polypharmacy, which can extend life expectancy and improve quality of life, versus problematic or inappropriate polypharmacy, which can increase the risk of drug interactions and adverse drug reactions (ADRs), as well as impairing adherence to medication and affecting quality of life.^6^ Various tools are available to identify and reduce polypharmacy as part of medication reviews. A helpful summary is provided by the Specialist Pharmacy Service.

Polypharmacy is causally linked with multimorbidity and is associated with significant morbidity and mortality, especially in older patients. There are excellent resources available for deprescribing from the British Geriatrics Society; however, polypharmacy is not restricted to older patients and deprescribing needs to be established as standard care by all clinicians.

The situation is compounded by a lack of clinical evidence; clinical trials rarely consider polypharmacy in the context of multimorbidity. There are numerous evidence-based guidelines for the treatment of single conditions (eg IBD), but personalised care requires guidelines on treatments for patients with multimorbidity.

## Health inequalities

Problematic polypharmacy tends to affect the most vulnerable in society, including those with multimorbidity, at the extremes of age, in lower socio-economic groups and marginalsied ethnic groups. These same patients are at increased risk of patient safety incidents compared to the general population. The COVID-19 pandemic shone a light on the scale of the health inequality challenges that we face. Core20PLUS5 is a national approach by NHS England to inform action to reduce healthcare inequalities. The NHS is starting to gather information on the diversity of the people affected by safety events, as well as those who deliver patient care. If we are able to view patient safety incidents through the eyes of the most disadvantaged, we may be able to make significant reductions in health inequalities.

## Education and training

**Fig. 2:**
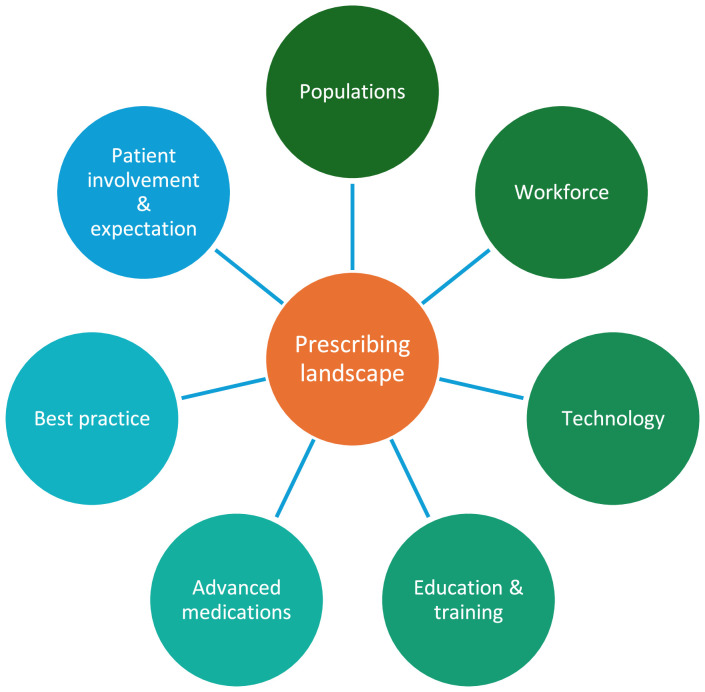
Challenges of an evolving prescribing landscape.

The management of polypharmacy as well as prescribing in modern, complex healthcare systems requires skilled multiprofessional working both in terms of medicines optimisation and reducing MEs. Currently, all newly qualified doctors in the UK are required to have completed the Prescribing Safety Assessment (PSA). There has been a reduction in the number of medication-related incident reports in the UK since its introduction. A recent independent review^7^ concluded that mandatory assessment of prescribing should remain a condition of practice for doctors in the UK, but recognised key challenges including:

• an inconsistent approach to the PSA in undergraduate education
• a lack of similar requirements for international medical graduates (IMGs)
• that current undergraduate training does not prepare doctors for the evolving prescribing landscape (see Fig. 2).

## Workforce and non-medical prescribing

A range of non-medical healthcare professionals (NMPs) can prescribe medicines for patients as either independent or supplementary prescribers. Independent prescribers are responsible and accountable for the assessment of patients and for decisions about the clinical management required, including prescribing. Supplementary prescribing is a partnership between an independent prescriber and a supplementary prescriber to implement an agreed clinical management plan for an individual patient.^8^

By 2026, the aim is for all newly registered pharmacists in the UK to be independent prescribers at qualification. Non-medical prescribing has been considered a natural extension to the role of a pharmacist; however, the uptake of non-medical prescribing by pharmacists to date has been slow. Despite a motivation to prescribe, increased job satisfaction and sense of professionalism, pharmacists who have trained as non-medical prescribers report feeling underprepared for the reality of unsupervised practice. The quality of training has been described as variable and the ability to then prescribe is dependent on local funding, access to medical records, time and support staff. Pharmacists report professional rivalry, with both support and resistance from members of the multiprofessional team.^9^ Current experience suggests that consistently and appropriately supporting NMPs to work within a multiprofessional team requires much more focus.

## Digital prescribing and artificial intelligence

It would be easy to think that artificial intelligence (AI) is the panacea for all things; however, our digital experience in healthcare has been mixed at best. Regarding medication safety, when electronic health records (EHR) and well-designed tools are available, it is possible to achieve up to a 45% reduction in unintentional discrepancies, improved patient–provider communication, optimisation of medication regimen and better patient medication adherence to treatment.^10^ There are obvious benefits to electronic medication systems (EMS), such as identifying drug interactions and supporting antimicrobial stewardship. EHR and EMS can also identify high-risk patients and high-risk medications, support deprescribing and reduce problematic polypharmacy.

However, implementation of EHR including EMS is a complex process that involves multiple stakeholders, all with different expectations and priorities. Successful deployment of an EMS is contingent on context, and the use of identical software can lead to very different results in different hospitals, in part because these systems are highly configurable at a local level. EMS are becoming increasingly complex, requiring iterative development and commitment to long-term investment. Despite the wide-scale adoption of EMS in hospitals around the world, the quality of evidence about their effectiveness in medication error and associated harm reduction is variable.

## Sustainability

Data published by the Royal Pharmaceutical Society (RPS) show that around 25% of NHS carbon emissions are from medicines. The majority of these emissions result from the manufacture, procurement, transport and use of medicines. Furthermore, there is medicines waste with unnecessary prescriptions, particularly on repeat prescriptions and hospital admissions secondary to medication-related adverse effects including ADRs, all of which contribute to carbon emissions from medicines.

There are now many initiatives looking at the environmental impact of all aspects of healthcare; for example, the work of Sustainability in Quality Improvement (SusQI). However, it remains true that most prescribers are not aware of the environmental impact of medicines. Guidelines do not routinely discuss environmental impact and there are no clear processes for choosing appropriate and efficacious medicines with the lowest environmental impact.

Recently, there has been a global increase in medicines shortages. The RPS will publish its review of the causes and impact of medicines shortages soon (and have already produced an open access webinar). However, early evidence suggests that shortages in the UK are primarily due to supply-based rather than demand-based causes. Further work continues with the Department of Health and Social Care to look at and improve the management of medicines shortages through Serious Shortage Protocols, which are issued when a medication is in short supply with the aim of reducing the risk of poor patient outcomes because of medicine shortages.

There are reasons to be optimistic, as advances in healthcare can support a more sustainable approach to medicines use; for example, as seen currently in the use of genomic and diagnostic characterisation in the treatment of certain cancers and HIV. It is also an opportunity to engage with patients to make informed choices and increase compliance.

## Conclusions

**Fig. 3:**
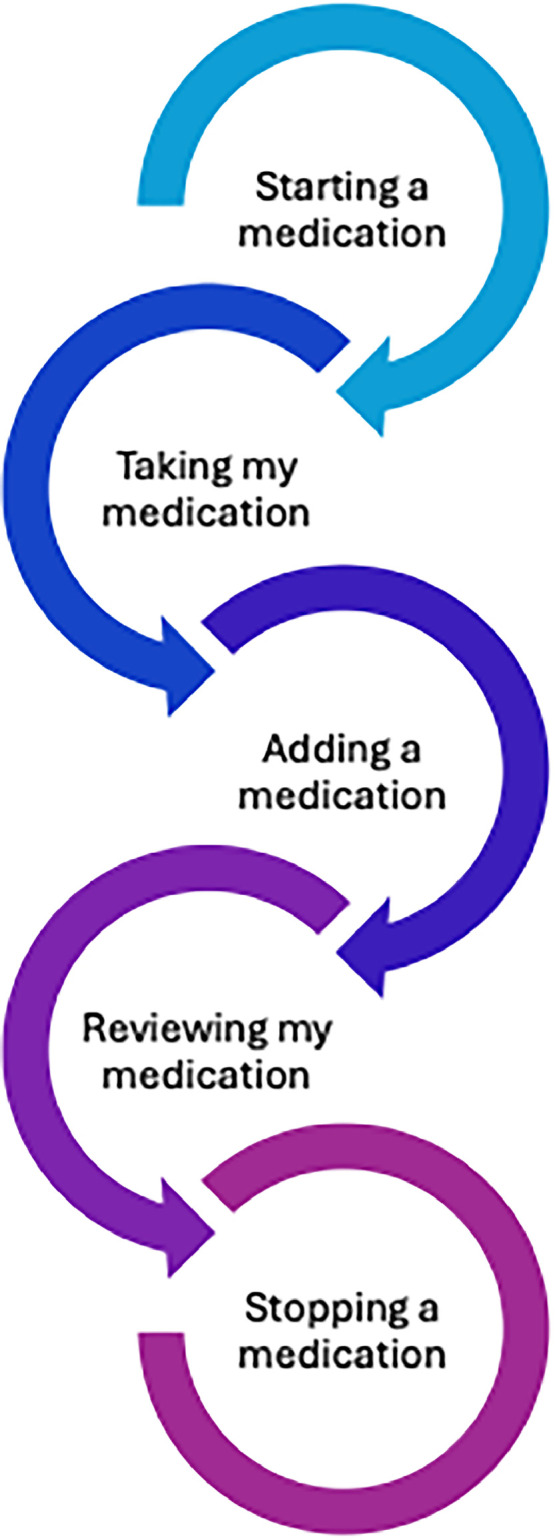
WHO five moments for medication safety.^11^

MEs are preventable and can be reduced with better processes and systems in place, as has been shown with the introduction of the PSA. However, we all have individual responsibility to improve medicines safety. When it comes to medicines management, we should stop and reflect at five key moments, as recommended by the WHO (see Fig. 3).

There are many improvement efforts underway that aim to reduce MEs, eg the improvement guide and resources published by the Royal College of Physicians focusing on medication safety at hospital discharge. NHS England has a dedicated Medicines Safety Improvement Programme and there is a similar programme in Scotland. However, the literature suggests that much more needs to be done to improve medicines safety in an increasingly complex and challenging healthcare landscape.

## CRediT authorship contribution statement

**Andrew Rochford:** Conceptualization, Data curation, Formal analysis, Funding acquisition, Investigation, Methodology, Project administration, Resources, Software, Supervision, Validation, Visualization, Writing – original draft, Writing – review & editing.

## Declaration of competing interest

The author declares that they have no known competing financial interests or personal relationships that could have appeared to influence the work reported in this paper.
